# Evaluation of day 5 versus day 6 blastocyst biopsy in preimplantation genetic testing: clinical and neonatal outcomes

**DOI:** 10.3389/fendo.2025.1544009

**Published:** 2025-06-16

**Authors:** Zhiming Li, Xiaowu Fang, Lizi Cheng, Zhanhui Ou, Junye Huo, Wenjuan Yu, Jieliang Li, Wanna Ke, Jiaqi Wu, Xiufeng Lin, Xiaojun Wen

**Affiliations:** ^1^ Reproductive Medicine Center, Boai Hospital of Zhongshan, Zhongshan, Guangdong, China; ^2^ The Second School of Clinical Medicine, Southern Medical University, Guangzhou, Guangdong, China

**Keywords:** preimplantation genetic testing, blastocyst biopsy, embryo quality, maternal age, clinical pregnancy outcomes, neonatal-perinatal outcomes

## Abstract

**Background:**

Preimplantation genetic testing (PGT) has emerged as a pivotal technique in assisted reproductive technology for enhancing success rates by identifying euploid embryos prior to transfer. The optimal timing for blastocyst biopsy during PGT remains controversial, with conflicting evidence regarding the clinical outcomes of day 5 (D5) versus day 6 (D6) biopsies, as well as neonatal and perinatal outcomes.

**Methods:**

This study involved a retrospective analysis of 3,647 biopsied blastocysts and 673 PGT-frozen embryo transfer (FET) cycles conducted at Zhongshan Boai Hospital between May 2019 and September 2024. Patients were categorized into D5 and D6 biopsy groups. The study comprised three components: (1) a comparison of chromosomal euploidy, mosaicism, and aneuploidy rates between the two groups, along with an assessment of clinical, neonatal, and perinatal outcomes in PGT-FET cycles; (2) stratification based on embryo quality to compare clinical, neonatal, and perinatal outcomes in PGT-FET cycles between the two groups; and (3) stratification according to maternal age to compare clinical, neonatal, and perinatal outcomes in PGT-FET cycles between the two groups.

**Results:**

The euploidy rate was significantly higher in D5 blastocysts compared to D6 blastocysts (47.53% vs. 32.38%, p < 0.01). In the PGT-FET cycles, the live birth rate in the D5 biopsy group was significantly higher than that in the D6 biopsy group (56.11% vs. 48.38%, p = 0.046); however, there were no significant differences in the clinical pregnancy rate, miscarriage rate, or neonatal outcome. Stratification by embryo quality revealed no significant differences in clinical pregnancy, live birth, or miscarriage rates for blastocysts of the same quality grade between the D5 and D6 biopsy groups. In the D5 biopsy group, variations in embryo quality did not affect clinical outcomes, whereas in the D6 biopsy group, high-quality blastocysts were associated with improved pregnancy and live birth rates. Age-stratified analysis showed similar clinical outcomes for PGT-FET in the D5 and D6 biopsy groups across different age groups.

**Conclusion:**

Compared to D6, D5 biopsied blastocysts demonstrated higher euploidy and live birth rates. Therefore, it is recommended to prioritize biopsy at D5 and to thaw blastocysts at D5 for transfer to achieve better clinical pregnancy and neonatal outcomes.

## 1 Introduction

In recent years, the development and application of preimplantation genetic testing (PGT) technology in reproductive medicine have become critical in enhancing the success rates of assisted reproductive technology. PGT technology employs genetic testing prior to embryo implantation to identify chromosomally normal embryos for transfer, thereby significantly improving clinical pregnancy rates and reducing the risks of miscarriage and transmission of genetic diseases. Currently, PGT technology is widely recognized and utilized in clinical practice, demonstrating clear advantages, particularly for women of advanced age, patients with recurrent miscarriages, couples with a genetic predisposition, and those who have experienced multiple *in vitro* fertilization failures (1, 2).

In PGT, embryo biopsy is a critical step for obtaining genetic material from the embryo. With advancements in embryo culture technology, the timing of biopsy has emerged as an important area of research. The developmental stage and quality of embryos are crucial factors influencing the success of frozen-thawed blastocyst transfer in achieving clinical pregnancy (3). Currently, many centers extend the blastocyst culture to day 6 (D6) to assess subsequent development and determine the necessity of embryo biopsy. Numerous studies have sought to compare clinical outcomes between blastocyst biopsies performed on day 5 (D5) and D6 in PGT cycles; however, the results have been inconsistent. Several studies comparing frozen-thawed PGT embryo transfers have indicated that chromosomally euploid D5 and D6 frozen-thawed blastocysts yield similar clinical pregnancy and live birth rates (4–8).

This finding indicates that regardless of the developmental stage of the blastocyst, there is no significant difference in the success rate of blastocyst transfer, provided that the embryo has been chromosomally screened through PGT and confirmed to be euploid. However, other studies identified certain advantages of D5 over D6 transfer in PGT cycles. Specifically, the chromosomal euploidy, pregnancy, implantation, and live birth rates of D5 blastocysts were significantly higher than those of D6 blastocysts, potentially related to factors such as embryo quality, patient age, endometrial condition, and body mass index (BMI) (9–14). Additionally, studies have reported that blastocyst transfer may increase the risk of prematurity, low birth weight, and macrosomia (15, 16); however, other studies have suggested that embryos transferred at the blastocyst stage do not significantly impact neonatal outcomes (17, 18).

Existing literature reveals conflicting findings regarding the outcomes of PGT, specifically concerning pregnancy, implantation, and live birth rates, as well as neonatal birth outcomes associated with D5 and D6 blastocyst biopsies. Currently, no consensus exists on this issue, underscoring the need for further studies to clarify the specific impact of D5 and D6 blastocyst biopsies on PGT results. Furthermore, few studies have assessed how the timing of blastocyst biopsy, along with factors such as patient age and embryo quality, influence clinical and neonatal outcomes in PGT cycles. Therefore, this retrospective study aims to compare the clinical pregnancy and neonatal outcomes of D5 and D6 PGT frozen embryo transfer (FET) cycles. The study considers varying embryo quality grades and patient ages to provide clearer guidance on the selection of embryo biopsy timing and embryo transfer.

## 2 Materials and methods

### 2.1 Ethics approval

This study was approved by the Reproductive Medicine Ethics Committee of Zhongshan Boai Hospital and the ethical approval number is KY-2025-004-04. Participants consisted of patients who underwent PGT biopsy and frozen-thawed embryo transfer cycles at Zhongshan Boai Hospital between May 2019 and September 2024. A total of 3647 blastocysts were biopsied, comprising 1,275 D5 blastocysts and 2372 D6 blastocysts. Additionally, there were 673 PGT frozen-thawed embryo transfer cycles, including 303 D5 frozen embryos and 370 D6 frozen embryos. All patients provided informed consent, which was approved by the local ethics committee, prior to both biopsy and embryo transfer.

### 2.2 Study design

**Figure 1:**
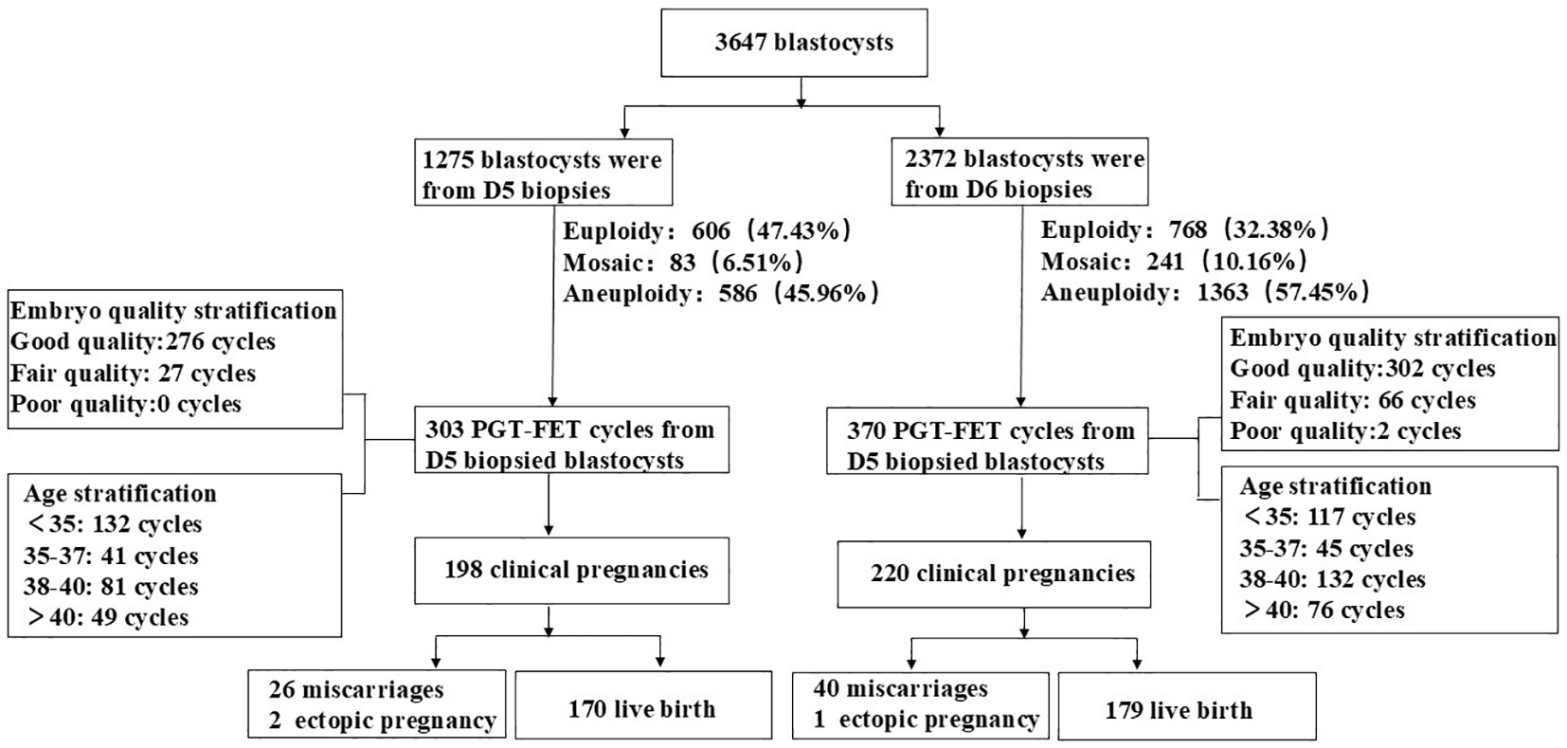
Study design flow chart.

Enrolled patients had common indications for PGT, excluding those undergoing PGT for monogenic disorders (PGT-M). The indications included older female patients (≥ 38 years), those with recurrent miscarriage (two or more occurrences), patients with repeated implantation failure (three or more instances), and individuals with chromosomal abnormalities, such as reciprocal translocations, Roche translocations, inversions, microdeletions, or duplications. This study primarily consisted of three retrospective analyses. In the first analysis, the patients were divided into two groups: D5 and D6 biopsy groups. The chromosomal euploidy, mosaicism, and aneuploidy rates were compared between the two groups. The second analysis involved a three-tier stratification based on embryo quality scores, categorizing embryos as good (AA/AB/BA), moderate (BB/AC), or poor (CA/BC/CB) and comparing the PGT results between the D5 and D6 biopsy groups across different embryo quality stratifications. The third analysis stratified patients according to age, specifically into four categories: < 35 years, 35–37 years, 38–40 years, and > 40 years, allowing for a comparison of PGT results between the D5 and D6 biopsy groups within these age stratifications. Study design flow chart is shown in **Figure 1**.

### 2.3 Oocyte retrieval, intracytoplasmic sperm injection, blastocyst culture, blastocyst scoring, blastocyst biopsy

Ovulation induction, intracytoplasmic sperm injection (ICSI), embryo culture, embryo biopsy, and embryo freezing were performed according to the standard protocols outlined in our previous study (19, 20). In summary, the ovulation induction regimen was tailored to the individual characteristics of the patient, including antagonist, micro-stimulation, short-acting, and long-acting protocols. When at least three follicles exceeded 18 mm in diameter, oocyte maturation was induced by intramuscular injection of human chorionic gonadotropin (HCG; Merck Serrano, Switzerland). After 36–38 h, vaginal ultrasound guidance was used to retrieve the oocytes. All mature oocytes underwent ICSI within 5–6 h post-retrieval. Fertilization status was assessed 16–18 h after ICSI, based on the presence of two pronuclei in the fertilized egg and expulsion of the second polar body. Embryos were cultured in G1-plus and G2-plus (Vitrolife, Sweden) sequential media. Embryonic development was monitored on D5 or D6, and the quality of blastocysts was evaluated according to the Gardner blastocyst scoring method to decide whether to proceed with biopsy (21). D5 or D6 blastocysts were deemed suitable for biopsy if they fulfilled the following criteria: a score of 3 or higher, with at least one score for either the inner cell mass (ICM) or trophoblast (TE) graded as B. The grades included AA, AB, AC, BA, BB, BC, CA, or CB. Biopsy was performed using a needle to aspirate trophoblast cells (4–10 cells) that protruded through the small openings in the zona pellucida, followed by laser-assisted cutting. Subsequently, the biopsied cells were washed in PBS droplets and transferred to 200 μl PCR reaction tubes containing cell lysis buffer, where they were frozen and stored at -20°C until chromosome detection. Embryos were frozen within 1 h post-biopsy to preserve their integrity.

### 2.4 PGT program

The PGT procedure has been described in detail in a previous study (20). In summary, the protocol utilized reagents from the ChromSwift kit (Yikon, China) to conduct whole-genome amplification via the multiple annealing and looping-based amplification cycles (MALBAC) method. Following amplification, the products were subjected to library construction, library concentration determination, and other subsequent steps, culminating in sequencing using an Illumina MiSeq high-throughput sequencer. An embryo was classified as euploid when the degree of chromosomal mosaicism was less than 20%. Chromosomal mosaicism was identified when the degree of mosaicism ranged from 20% to 80%. Chromosomal aneuploidy was diagnosed when the degree of mosaicism exceeded 80% (22).

### 2.5 Endometrial preparation, embryo transfer, and follow-up

The preparation of the endometrium was tailored to the patient’s specific condition, which included natural, artificial, or stimulation cycles. Euploid embryos exhibiting the highest morphological grades were selected for thawing and were transferred into the uterine cavity 4–5 h post-thawing. Serum β-HCG levels were measured on the 14th day following transfer. A vaginal B-ultrasound examination performed on the 35th day post-transfer confirmed clinical pregnancy by visualizing a fetal sac within the uterus, an embryo contained in the sac, and a heartbeat. Miscarriage was defined as fetal loss occurring before 20 weeks of gestation. Live birth was classified as the delivery of a viable baby after 24 weeks of gestation, with delivery methods encompassing both cesarean section and natural delivery. Premature birth was defined as the delivery of a viable fetus at less than 37 weeks of gestation. High and low birth weights were defined as ≥ 4000 g and < 2500 g, respectively.

### 2.6 Result measurement calculation

The clinical pregnancy rate was calculated as the number of clinical pregnancy cycles divided by the number of transplantation cycles multiplied by 100%. The miscarriage rate was determined by dividing the number of abortion cycles by the number of clinical pregnancy cycles and multiplying by 100%. The live birth rate was obtained by dividing the number of cycles resulting in active infant delivery by the number of transfer cycles and multiplying by 100%. The euploidy rate was defined as the number of euploid embryos divided by the number of embryos tested by PGT, multiplied by 100%. The mosaic rate was defined as the number of chimeric embryos divided by the number of embryos tested by PGT, multiplied by 100%. Finally, the aneuploidy rate was calculated by dividing the number of aneuploid embryos by the total number of embryos detected by PGT and multiplying by 100%.

### 2.7 Statistics

Statistical analysis of the collected data was performed using SPSS version 27.0 (IBM Corporation, USA). Categorical variables are presented as counts and percentages, with the chi-square test or Fisher’s exact test employed for comparative analysis. The assessed indicators included the proportions of infertility types as well as fertilization rate, chromosomal euploidy, mosaicism, aneuploidy, clinical pregnancy, miscarriage, live birth, premature birth, high birth weight, and low birth weight rates. The t-test was used for statistical analysis to evaluate the differences in variables between groups. The variables analyzed included number of miscarriages, female age, female BMI (body mass index), thickness of endometrium, length of infertility history, number of abortions, basal hormone levels, number of oocytes retrieved, number of MII (Metaphase II) oocytes, gestation days, birth length, and infant birth weight. Statistical significance was set at p < 0.05.

## 3 Results

### 3.1 Comparison of basic clinical features and PGT results between the D5 and D6 TE biopsy groups

A total of 3,647 blastocysts were subjected to TE biopsy, comprising1275 D5 blastocysts and 2,372 D6 blastocysts. **Table 1** presents the characteristics of patients in the D5 and D6 blastocyst groups that underwent freeze-thaw transfer. The data indicated that both groups exhibited similar characteristics. Specifically, there were no statistically significant differences between the groups regarding age, BMI, endometrial thickness, infertility type, infertility duration, number of miscarriages, basal hormone levels, or anti-Müllerian hormone (AMH) levels. **Table 2** displays the PGT results for both the D5 and D6 blastocyst groups in the context of FET. Significant differences were observed in the rates of chromosomal euploidy, mosaicism, and aneuploidy between the two groups. The chromosomal euploidy rate in the D5 TE biopsy group was significantly higher than that in the D6 TE biopsy group (47.53% vs. 32.38%, p < 0.01), whereas the rates of chromosomal mosaicism and aneuploidy in the D5 TE biopsy group were significantly lower than those in the D6 TE biopsy group (6.51% vs. 10.61%, p < 0.01; 45.96% vs. 57.46%, p < 0.01).

**Table 1:** Based on maternal characteristics of D5 and D6 trophoectoderm biopsies.

Characteristics	D5	D6	P
Female age (years, average ± SD)	35.30±4.88	36.65±4.68	0.158
Female BMI (kg/m2, average ± SD)	22.24±2.94	22.63±3.51	0.207
Thickness of endometrium (mm, average ± SD)	9.81±3.35	9.59±1.83	0.324
Types of infertility			
Primary infertility (n ,%)	63(20.8%)	57(15.4%)	0.069
Secondary infertility (n,%)	240(79.2%)	313(84.6%)	
Length of infertility history(years, average ± SD)	4.19±3.15	4.25±3.38	0.533
Number of abortions(average ± SD)	1.59±1.38	1.51±1.49	0.698
Basal sexual hormone			
FSH (IU/L)	7.77±2.23	7.74±2.03	0.677
LH (IU/L)	4.80±3.09	4.46±2.27	0.196
E2 (pg/mL)	52.59±157.39	52.11±181.15	0.742
P(ng/mL)	0.61±0.61	0.70±1.24	0.177
AMH(ng/mL)	3.65±2.41	3.08±2.06	0.051
Number of oocytes retrieved (average ± SD)	11.14±5.84	10.95±6.25	0.126
Number of MII oocytes (average ± SD)	9.86±5.41	9.34±5.49	0.668
Fertilization rate, n (%)	91.73% (2741/2988)	92.65% (3202/3456)	0.170

**Table 2:** Comparison of PGT results from D5 and D6 trophoectoderm biopsies.

Item	D5	D6	P
No. of blastocysts biopsied(n)	1275	2372	–
No. of blastocysts in chromosome euploidy(n,%)	606 (47.53%)	768 (32.38%)	<0.01
No. of blastocysts in chromosome mosaic(n,%)	83 (6.51%)	241 (10.16%)	<0.01
No. of blastocysts in chromosome aneuploidy(n,%)	586 (45.96%)	1363 (57.46%)	<0.01

### 3.2 Comparison of clinical and neonatal-perinatal outcomes between D5 and D6 TE biopsy groups during the PGT-FET cycle

A total of 673 PGT-FET cycles were conducted, with one euploid embryo transferred per cycle. Of these, 303 cycles were in the D5 TE biopsy group, and 370 were in the D6 TE biopsy group (**Table 3**). Although neither the clinical pregnancy rate nor the miscarriage rate was significantly different between the two groups, the D5 TE biopsy group exhibited a higher clinical pregnancy rate (65.35% vs. 59.46%, p = 0.057) and a lower miscarriage rate (13.13% vs. 18.18%, p = 0.157) compared to the D6 TE biopsy group. The live birth rate in the D5 TE biopsy group was significantly higher than that in the D6 TE biopsy group (56.11% vs. 48.38%, p = 0.046). Follow-up results indicated no statistically significant differences between the two groups regarding gestational days, birth length, birth weight, sex ratio, high birth weight rate, or low birth weight rate. Data analysis revealed that the preterm birth rate in the D5 TE biopsy group was slightly lower than that in the D6 TE biopsy group, although this difference was not statistically significant. Additionally, one birth defect was reported in each group: esophageal atresia in the D5 TE biopsy group and cleft lip and palate in the D6 TE biopsy group.

**Table 3:** Comparison of PGT-FET transplantation and neonatal clinical outcomes for D5 and D6 biopsies.

Item	D5	D6	P
FET cycles,n	303	370	–
Clinical pregnancy rate, (%,n)	65.35%(198/303)	59.46%(220/370)	0.057
Miscarriage rate, (%,n)	13.13%(26/198)	18.18%(40/220)	0.157
Live birth rate, (%,n)	56.11%(170/303)	48.38%(179/370)	0.046*
Gestation days (average ± SD)	268.26±13.10	267.80±15.45	0.516
Birth Length (cm, average ± SD)	48.79±2.38	48.60±2.86	0.620
Birth weight(g, average ± SD)	3172.49±506.00	3143.83±551.69	0.686
Birth sex, male (%,n)	58.82%(100/170)	58.66%(105/179)	0.975
Birth sex, female (%,n)	41.18%(70/170)	41.34%(74/179)	
premature birth, (%,n)	12.94%(22/170)	16.76%(30/179)	0.317
High birth weight (%,n)	4.12%(7/170)	2.79%(5/179)	0.479
Low birth weight (%,n)	7.65%(13/170)	8.94%(16/179)	0.662
Birth defect (%,n)	0.59%(1/170)	0.56%(1/179)	–

### 3.3 Clinical and neonatal-perinatal outcomes in the D5 and D6 TE biopsy groups stratified by embryo quality during the PGT-FET cycle

To further investigate the effect of blastocyst development on reproductive outcomes, we categorized blastocysts into three groups based on their scoring levels: well-developed (AA/AB/BA; D5 n = 276, D6 n = 302), moderately developed (BB/AC; D5 n = 27, D6 n = 66), and poorly developed (CA/BC/CB; D5 n = 0, D6 n = 2). Due to the limited number of poor blastocysts in the PGT-FET cycle, they were excluded from the statistical analysis. As illustrated in **Table 4**, within the same embryo quality grade, both the D5 and D6 TE biopsy groups demonstrated similar PGT-FET clinical outcomes (including clinical pregnancy, miscarriage, and live birth rates) and neonatal-perinatal outcomes (gestational days, birth length, birth weight, and sex ratio).

**Table 4:** Comparison of PGT-FET transplantation and neonatal clinical outcomes based on embryo quality stratification by D5 and D6 biopsies.

Ttem	AA/AB/BA			BB/AC			P^a^	P^b^
	D5^a^	D6^b^	P	D5 ^a^	D6^b^	P		
FET cycles,n	276	302	–	27	66	–	–	
Clinical pregnancy rate, (%,n)	66.30% (183/276)	63.91% (193/302)	0.546	55.56% (15/27)	40.91% (27/66)	0.198	0.263	<0.01*
Miscarriage rate, (%,n)	12.02% (22/183)	16.06% (31/193)	0.260	26.67% (4/15)	33.33% (9/27)	0.290	0.116	0.029*
Live birth rate, (%,n)	57.61% (159/276)	53.31% (161/302)	0.299	40.74% (11/27)	27.27% (18/66)	0.203	0.092	<0.01*
Gestation days (average ± SD)	268.33±13.33	267.74±16.08	0.395	266.00±10.11	238.39±8.01	0.109	0.881	0.300
Birth Length (cm, average ± SD)	48.75±2.42	48.62±2.90	0.745	49.45±1.69	48.44±2.45	0.275	0.374	0.915
Birth weight(g, average ± SD)	3165.85±509.47	3146..18±560.90	0.664	3232.27±478.00	3143.33±485.50	0.956	0.856	0.962
Birth sex, male (%,n)	61.01% (97/159)	58.39% (94/161)	0.633	27.27% (3/11)	61.11% (11/18)	0.166	0.053	0.824
Birth sex, female (%,n)	38.99% (62/159)	41.61% (67/161)		72.73% (8/11)	38.89% (7/18)	

P^a^ represents a comparison of PGT-FET clinical and neonatal outcomes in the D5 TE biopsy group with well-developed (AA/AB/BA) and moderately developed (BB/AC) blastocysts.

P^b^ represents a comparison of PGT-FET clinical and neonatal outcomes in the D6 TE biopsy group with well-developed (AA/AB/BA) and moderately developed (BB/AC) blastocysts.

This finding suggests that for blastocysts of the same quality, the developmental duration does not influence reproductive outcomes. Among D5 blastocysts, there was no statistically significant difference in PGT-FET clinical or neonatal outcomes between well-developed and moderately developed blastocysts. In contrast, among D6 blastocysts, the clinical pregnancy (p < 0.001) and live birth rates (p < 0.001) were significantly higher for well-developed blastocysts than for moderately developed blastocysts, whereas the miscarriage rate (p = 0.029) was significantly lower. No statistically significant differences were observed between the two groups in terms of neonatal outcomes.

### 3.4 Clinical and neonatal-perinatal outcomes in the D5 and D6 TE biopsy groups stratified by maternal age during the PGT-FET cycle.

As shown in **Table 5**, for female patients within the same age group, there were no statistically significant differences in the clinical pregnancy, miscarriage, or live birth rates between the D5 and D6 TE biopsy groups. Regarding neonatal outcomes, follow-up data indicated that in the group aged > 40 years, the number of gestational days, birth length, and birth weight in the D5 TE biopsy group were significantly lower than those in the D6 TE biopsy group. Conversely, in the 35–37-year-old group, the birth length in the D5 TE biopsy group was significantly greater than that in the D6 TE biopsy group. Aside from these findings, no significant differences were observed in other indicators.

**Table 5:** Comparison of PGT-FET transplantation and neonatal clinical outcomes based on age-stratified by D5 and D6 biopsies.

Item	<35			35-37			38-40			> 40		
	D5	D6	P	D5	D6	P	D5	D6	P	D5	D6	P
FET cycles,n	132	117	–	41	45	–	81	132	–	49	76	–
Clinical pregnancy rate, (%,n)	67.42% (89/132)	58.97% (69/117)	0.528	68.29% (28/41)	55.56% (25/45)	0.225	62.96% (51/81)	60.61% (80/132)	0.731	61.22% (30/49)	60.53% (46/76)	0.938
Miscarriage rate, (%,n)	6.74% (6/89)	11.97% (14/117)	0.210	25.00% (7/28)	12.00% (3/25)	0.243	15.69% (8/51)	17.50% (14/80)	0.787	16.67% (5/30)	19.57% (9/46)	0.750
Live birth rate, (%,n)	62.88% (83/132)	47.01% (55/117)	0.200	51.22% (21/41)	48.89% (22/45)	0.829	50.62% (41/81)	50.00% (66/132)	0.930	51.02% (25/49)	47.37% (36/76)	0.690
Gestation days (average ± SD)	269.83 ±12.52	270.65 ±9.89	0.979	266.67 ±9.88	267.86 ±18.28	0.140	265.39 ±13.13	264.24 ±19.72	0.328	264.07 ±17.09	269.94 ±9.96	0.013*
Birth Length (cm, average ± SD)	48.69 ±2.34	49.25 ±1.95	0.475	49.10 ±1.41	48.18 ±3.88	0.017*	48.90 ±2.64	47.94 ±3.49	0.950	48.72 ±2.78	49.08 ±1.54	0.032*
Birth weight (g, average ± SD)	3138.92 ±449.92	3212.82 ±473.25	0.325	3363.57 ±406.88	3086.59 ±720.69	0.079	3149.02 ±566.98	3024.85 ±606.08	0.669	3146.00 ±634.84	3301.81 ±389.16	0.028*
Birth sex, male (%,n)	55.42% (46/83)	61.82% (34/55)	0.135	66.67% (14/21)	59.09% (13/22)	0.607	65.85% (27/41)	57.58% (38/66)	0.394	52.00% (13/25)	55.56% (20/36)	0.784
Birth sex, female (%,n)	44.58% (37/83)	38.18% (21/55)		33.33% (7/21)	40.91% (9/22)		34.15% (14/41)	42.42% (28/66)		48.00% (12/25)	44.44% (16/36)	

## 4 Discussion

This study retrospectively analyzed the chromosomal euploidy status of 3,647 biopsied blastocysts and evaluated the impact of blastocyst development days, blastocyst quality, and female patient age on clinical pregnancy outcomes and neonatal health using follow-up data from 673 PGT-FET cycles. The findings indicated that the chromosomal euploidy rate of D5 blastocysts was significantly higher than that of D6 blastocysts. Furthermore, the analysis of follow-up data revealed that the transfer of D5 euploid blastocysts significantly increased the live birth rate compared to the transfer of D6 euploid embryos. However, no significant differences were observed in the clinical pregnancy rates, miscarriage rates, or neonatal and perinatal outcomes.

The optimal timing of frozen embryo transfer has been a contentious subject. However, the evidence regarding the differences between D5 and D6 euploid blastocyst transfers remains inconsistent. Variability in blastocyst culture strategies and methodologies employed by different centers has resulted in inconsistent findings concerning the relationship between chromosomal abnormalities and the days of embryonic development. Taylor et al. conducted a retrospective analysis of 421 D5 and 413 D6 blastocysts and found that the euploidy rate of D5 blastocysts was significantly higher than that of D6 blastocysts, suggesting that slower-growing blastocysts may exhibit a higher aneuploidy rate (23). Conversely, Yang et al. reported no significant differences in chromosomal euploidy rates between D5 and D6 blastocysts, indicating comparable developmental potential (7). More recently, Wu et al.found that regardless of whether patients were older or younger than 38 years, there were no significant differences between D5 and D6 blastocysts in the PGT-A cycle in terms of chromosomal euploid, aneuploid, or mosaic rates (24). Our study aligns with the findings of Taylor et al., demonstrating that the aneuploidy rate in D6 blastocysts (57.46%) is significantly higher than that in D5 blastocysts (45.96%). This discrepancy may contribute to the observed superiority of D5 blastocyst transfers over D6 blastocyst transfers in non-PGT-FET cycles (11, 13, 25).

The comparison of pregnancy outcomes between D5 and D6 euploid blastocysts remains controversial. Toukhy et al. and Kaye et al. reported no significant differences in clinical pregnancy and live birth rates between transfers of high-quality D5 and D6 blastocysts during FET cycles (5, 6). Similarly, Wu et al., in a retrospective analysis of 527 PGT-FET cycles, found no significant differences in the pregnancy (D5:53.4%, D6:52.4%, p = 0.838), live birth (D5:46.1%, D6:44.1%, p = 0.682), and miscarriage rates (D5:13.7%, D6:16.0%, p = 0.858) of transferred D5 and D6 euploid blastocysts (24). However, a retrospective analysis by Irani et al. involving 701 cycles of frozen-thawed haploid blastocyst transfer indicated that the live birth rate of D5 euploid blastocysts was significantly higher than that of D6 euploid blastocysts transferred in PGT-FET cycle (26). This observation aligns with the findings of Chen et al., which demonstrated higher implantation rates (D5: 67.9% vs. D6: 46.6%, p < 0.001) and live birth rates (D5: 61.6% vs. D6: 39.2%, p < 0.001) for D5 blastocysts, although there was no significant difference in miscarriage rates (27). Additionally, Yu et al. found that D5 blastocysts exhibited higher success rates and lower miscarriage rates after transfer compared to D6 blastocysts (28). In our study, we found that the live birth rate in the D5 biopsy group was significantly higher than that in the D6 biopsy group (D5: 56.11%, D6: 48.38%, p = 0.046). The clinical pregnancy rate in the D5 biopsy group also exhibited a favorable trend (D5: 65.35%, D6: 59.46%, p = 0.057), although this difference was not statistically significant. The miscarriage rates were comparable between the two groups. Possible explanations for these outcomes include the fact that D5 blastocysts represent faster-developing embryos, which may possess more optimal metabolic and epigenetic states (e.g., mitochondrial function, DNA methylation). In contrast, the delayed development of D6 blastocysts may indicate a reduced potential for implantation or placental formation, potentially leading to covert losses during mid-to-late pregnancy (e.g., placental insufficiency). This may ultimately manifest as differences in live birth rates rather than early miscarriage rates (29–31). Although the differences in clinical pregnancy and miscarriage rates between the D5 and D6 biopsy groups did not achieve statistical significance, the absolute differences may not have been detected due to sample size limitations. The live birth rate, as a cumulative endpoint, magnifies the impact of these differences. Furthermore, D5 blastocysts are closer to the timing of blastocyst entry into the uterine cavity in a natural cycle, whereas endometrial receptivity may be slightly altered by the time D6 blastocyst transfer, potentially affecting pregnancy maintenance. This is consistent with the findings of Irani et al., which indicate that the ongoing pregnancy rate is higher for D5 blastocysts, although no differences were observed in early pregnancy indicators (26). Notably, stratified analysis by embryo quality showed no significant difference in clinical outcomes between high-quality (AA/AB/BA) D6 and D5 blastocysts. However, the live birth rate of moderate -quality D6 blastocysts was significantly lower than that of D5 blastocysts (27.27% vs. 56.11%), a difference that may account for the overall advantage of D5 blastocysts group in the comprehensive analysis.

Embryo quality is a critical factor influencing the success of FET cycles (3). Therefore, we performed a stratified analysis of embryo quality in blastocysts from both the D5 and D6 biopsy groups. The findings indicated that, among blastocysts of the same grade, the clinical pregnancy, live birth, and miscarriage rates were similar between the D5 and D6 biopsy groups, aligning with previous studies (32, 33). We discovered for the first time that among D5 blastocysts, there was no significant difference in clinical pregnancy, live birth, or miscarriage rates between blastocyst groups of different grades. However, for D6 blastocysts, the clinical pregnancy rate (good: 63.91% vs. medium: 40.91%, p < 0.01) and live birth rate (good: 53.31% vs. medium: 27.27%, p < 0.01) were significantly higher than those of medium quality blastocysts, whereas the miscarriage rate (good: 16.06% vs medium: 33.33%, p = 0.029) was significantly lower. Maternal age is another critical factor influencing pregnancy outcomes; however, our findings indicated that the clinical outcomes of PGT-FET on D5 and D6 were comparable, regardless of maternal age.

There are relatively few studies comparing neonatal-perinatal outcomes between D5 and D6 blastocysts, and variations in embryonic developmental rates may influence these outcomes. Our findings indicate that the D5 and D6 biopsy groups exhibited similar outcomes in terms of gestational age, length, weight, and sex ratio, suggesting that the morphology and developmental speed of the embryo are not correlated with neonatal outcomes, which is consistent with the findings of Shi et al. (34) and Chen et al. (27). However, some studies have identified that the only significant difference in perinatal outcomes between the D5 and D6 groups was live birth weight. Specifically, Wu et al. and Sardana et al. observed higher birth weights in the D5 group, a trend also reflected in our data, although the difference was not statistically significant (24, 32). Conversely, other studies have concluded that D6 frozen-thawed blastocyst transfer is associated with higher birth weights (34). The discrepancies in perinatal weight between D5 and D6 blastocyst transfers may be attributed to factors such as prolonged *in vitro* culture, placental development, and gestational duration, as noted by Zeng et al. (24, 35). Our findings revealed that for maternal age of 35–37 years, the lengths of newborns in the D5 biopsy group were significantly greater compared to the D6 biopsy group. Conversely, for maternal age > 40 years, the lengths of newborns in the D5 biopsy group were significantly lower than those in the D6 biopsy group.

This study has certain limitations. First, in the stratified analysis of embryo quality, the sample size of poor-quality embryos (CA/BC/CB) was insufficient for statistical inclusion. Second, while this study offers evidence supporting embryo selection in PGT-FET cycles, its findings may not be applicable to fresh PGT embryo transfer cycles.

In conclusion, variations in embryo culture systems, culture strategies, freezing and thawing methods, and embryo transfer protocols employed by different centers may have contributed to the observed discrepancies in study outcomes. Our study revealed that both euploidy and live birth rates were higher in the D5 biopsy group compared to the D6 group, supporting D5 blastocysts as the preferred choice for embryo transfer. However, stratified analysis showed that high-quality (AA/AB/BA) D6 blastocysts had comparable clinical outcomes to D5 blastocysts. Therefore, in cases where D5 blastocysts are limited, high-quality D6 blastocysts remain a viable alternative, provided that strict morphological assessment is applied.

## Data Availability

The data that support the findings of this study have been deposited into CNGB Sequence Archive (CNSA) of China National GeneBank DataBase (CNGBdb) with accession number CNP0007494.
